# Best practice considerations on the assessment of robotic assisted surgical systems: results from an international consensus expert panel

**DOI:** 10.1017/S0266462323000314

**Published:** 2023-06-05

**Authors:** Jamie Erskine, Payam Abrishami, Richard Charter, Americo Cicchetti, Richard Culbertson, Eliney Faria, Jo Carol Hiatt, Jim Khan, Guy Maddern, Anita Patel, Koon Ho Rha, Paresh Shah, Prasanna Sooriakumaran, Scott Tackett, Giuseppe Turchetti, Anastasia Chalkidou

**Affiliations:** 1 Alira Health, London, UK; 2 Erasmus School of Health Policy and Management, National Health Care Institute, Rotterdam, The Netherlands; 3 HTAi – Health Technology Assessment International, Edmonton, AB, Canada; 4 Department of Economics and Business Management, Università Cattolica del Sacro Cuore, Milano, Italy; 5 Louisiana State University Health Sciences Centre, New Orleans, LA, USA; 6 Department of Urology, Hospital Felicio Rocho, Belo Horizonte, Brazil; 7 Medical Device Innovation Consortium, Arlington, VA, USA; 8 Portsmouth Hospitals NHS Trust, University of Portsmouth, Portsmouth, UK; 9 Discipline of Surgery, University of Adelaide, Adelaide, SA, Australia; 10 Ipsos, London, UK; 11 University of East Anglia, Norwich, UK; 12 Yonsei University Medical School, Seoul, South Korea; 13 NYU Langone, New York City, NY, USA; 14 Lerner College of Medicine, Cleveland Clinic, London, UK; 15 Department of Urology, University College London Hospital, London, UK; 16 Nuffield Department of Surgical Sciences, University of Oxford, Oxford, UK; 17 Intuitive Surgical, Sunnyvale, CA, USA; 18 Management Institute, Scuola Superiore di Studi Universitari e di Perfezionamento Sant’Anna, Pisa, Italy; 19 National Institute of Health and Care Excellence, London, UK

**Keywords:** Robotic surgery, Health technology assessment, health economics, robotic assisted surgery, consensus conference

## Abstract

**Background:**

Health technology assessments (HTAs) of robotic assisted surgery (RAS) face several challenges in assessing the value of robotic surgical platforms. As a result of using different assessment methods, previous HTAs have reached different conclusions when evaluating RAS. While the number of available systems and surgical procedures is rapidly growing, existing frameworks for assessing MedTech provide a starting point, but specific considerations are needed for HTAs of RAS to ensure consistent results. This work aimed to discuss different approaches and produce guidance on evaluating RAS.

**Methods:**

A consensus conference research methodology was adopted. A panel of 14 experts was assembled with international experience and representing relevant stakeholders: clinicians, health economists, HTA practitioners, policy makers, and industry. A review of previous HTAs was performed and seven key themes were extracted from the literature for consideration. Over five meetings, the panel discussed the key themes and formulated consensus statements.

**Results:**

A total of ninety-eight previous HTAs were identified from twenty-five total countries. The seven key themes were evidence inclusion and exclusion, patient- and clinician-reported outcomes, the learning curve, allocation of costs, appropriate time horizons, economic analysis methods, and robotic ecosystem/wider benefits.

**Conclusions:**

Robotic surgical platforms are tools, not therapies. Their value varies according to context and should be considered across therapeutic areas and stakeholders. The principles set out in this paper should help HTA bodies at all levels to evaluate RAS. This work may serve as a case study for rapidly developing areas in MedTech that require particular consideration for HTAs.

## Introduction

Robotic assisted surgery (RAS) has been increasingly adopted in clinical practice globally over the past two decades. Since 2000, an increasing number of robotic platforms have been developed, with over 150 companies operating in the field today (1). As the technology develops further and demand increases, health technology assessment (HTA) agencies face challenges when evaluating the utility of robotic systems.

Difficulties arise when HTA bodies use different methodologies or different inclusion and exclusion criteria within assessments (see Supplementary Table 1). Previous HTAs have shown that clinical outcomes are superior when compared to open surgery; however, most HTAs conclude that results are similar or marginally improved when compared to laparoscopic surgery (2–5). Quality assessments of the evidence also consistently report low-to-moderate quality and moderate-to-high risk of bias (2;5–7). The IDEAL framework (8) for surgical innovation suggests using CONSORT (9) for conducting randomized controlled trials (RCTs); however, RCTs of RAS are commonly concluded to be difficult to perform due to impracticalities in blinding, randomization, and recruitment (6;10), particularly when procedures are already well adopted (11). Furthermore, given the complex interrelations of the institutional context in which platforms are used, RCTs may not reflect the way in which the system will be used in real-life clinical settings. The IDEAL framework encourages real-world evidence (RWE) for long-term monitoring but in practice, observational studies and RWE are often deprioritized or considered insufficient to inform decision making in HTAs (5–7). Abrishami et al. (12) concluded that both in research and in clinical experience, there are disagreements on everything from “study results, to designs, methods, and purposes of studies, right down to what the very concept of ‘value’ constitutes” for the patient, surgeon, and healthcare system as a whole.

Previous reports, such as a 2019 HTA on robotic thoracic surgery from Wales (13), conclude that patient and clinician-relevant outcomes would be welcomed, but there is a lack of appropriate data (5).

As with other medical devices, outcomes of RAS are associated with end-user experience, and thus learning curve effects have an impact. HTAs have attempted to address impacts of learning curve effects in various ways, recognizing that these effects may confound results, but there remains a lack of standardized consideration in HTAs (2;3;4;14;15). Economic analyses have also been heterogeneous and have resulted in a wide range of incremental cost-effectiveness ratios (ICERs) (see Supplementary Table 2). Evaluations have arrived at vastly different conclusions, due to their use of different time horizons, discount rates, allocations of capital costs, procedure volumes, and system lifespans (see Supplementary Table 2). ICERs have ranged between $5.2 million (CAD) per quality-adjusted life-year (QALY) and $25,704 (CAD) per QALY for the same platform and the same procedure (4;16).

Furthermore, the robot itself is often part of a larger ecosystem of services that traditionally are not captured in HTAs. Added value of improved surgeon ergonomics and longevity, faster learning curves and ease of use, training, simulation, and digital services bring other value propositions not often considered. The value of these additional benefits also depends on the perspective of the HTA, whether national or hospital based.

Although existing frameworks for HTA (17) do provide some guidance on topics discussed above, there are additional considerations for RAS that have not yet been reported to our knowledge. EUnetHTA’s HTA core model (17) states that “Agreement on methodological criteria for non-randomized trials and observational studies is considerably less well-developed,” than for RCTs. The National Institute for Health and Care Excellence recently published their framework for RWE which provides guidance on the planning, conduct, and reporting for real-world studies (18). It notes that the increased focus on lifecycle evaluation of technology requires nonrandomized studies, and this certainly pertains to RAS.

EUnetHTA also provides guidance on patient-reported outcomes (PROs) (17), and most HTAs recognize the importance of including the patient perspective, but the extent and influence of such information vary between agencies (19;20). There is even less guidance on how benefit to the surgeon and surgical team should be considered. Self-reported instruments and tools have been used in the literature (21–33) but have not been considered by many HTAs. Furthermore, guidance for economic analysis will vary across geographies and guidance on learning curve is not often explicitly considered (34;35).

Many of these challenges faced by HTAs stem from the fact that platforms have been evaluated in the context of therapy, similarly to how pharmaceuticals are evaluated. This has resulted in HTAs across the globe arriving at different conclusions. As RAS continues to be adopted, further guidance is needed to assess it more appropriately. This work aimed to address these issues by assembling an expert panel to discuss the topic and produce guidance for assessing RAS.

## Methods

The HTAi Medical Device Interest Group (MDIG) was established in 2019. A major aim of the group is to develop research on HTA methods in medical devices, diagnostics, and digital health technology. Given the methodological challenges with HTA for RAS, the group considered that there was significant need for expert input in the development of recommendations for HTA practitioners in the assessment of RAS. A consensus development research methodology was considered most appropriate for this purpose (36).

A study steering committee was developed with MDIG leadership serving as co-chairs of the expert panel. The committee developed a shortlist of potential expert contributors. All experts on the shortlist were invited to participate and asked for recommendations from their network to join the panel. The aim was to finalize a panel of 11–15 panellists with global diversity and representing relevant stakeholders: clinicians, health economists, HTA practitioners, policy makers, methodologists, and industry (Table 1).

**Table 1.** List of experts and affiliations

Expert	Affiliations	Country
Anastasia Chalkidou – CHAIR	NICE/HTAi	United Kingdom
Guy Maddern	University of Adelaide	Australia
Paresh C. Shah	NYU Langone Health System	United States of America
Giuseppe Turchetti	Scuola Superiore Sant’Anna	Italy
Americo Cicchetti	Università Cattolica del Sacro Cuore	Italy
Jo Carol Hiatt	MDIC	United States of America
Payam Abrishami	ZorgInstituut	Netherlands
Eliney Faria	Hospital Felicio Rocho	Brazil
Scott Tackett	Intuitive Surgical	United States of America
Jim Khan	Portsmouth University Hospital NHS Trust	United Kingdom
Prasanna Sooriakumaran	Cleveland Clinic	United Kingdom
Richard Culbertson	LSU School of Public Health	United States
Koon Ho Rha	Yonsei University Medical School	South Korea
Anita Patel	IPSOS, University of East Anglia	United Kingdom

HTAi, Health Technology Assessment International; LSU, Louisiana State University; MDIC, Medical Device Innovation Consortium; NICE, National Institute for Health and Care Excellence; NYU, New York University.

The panel met five times in total between January and June 2022. The first meeting introduced the panel members to each other, and the steering committee introduced the aims of the work. A Population, Intervention, Comparator, Outcomes (PICO) statement was agreed upon by the panel (see Table 2). The scope of the project was limited to soft-tissue surgeries, as most past HTAs focused on these surgeries.

**Table 2.** Final PICOT statement as agreed by the panel

Population	Patients undergoing: Soft-tissue surgery/laparoscopy Prostatectomy Hernia repair Hysterectomy Rectal resection Partial nephrectomy Cholecystectomy Colectomy
Intervention	Robotic assisted surgery systems
Comparator	Open surgery Nonrobotic laparoscopic surgery
Outcomes	Survival/mortality Morbidity/functional complications Disease-specific effectiveness measures Procedure-specific effectiveness measures Patient-reported outcomes Resource use Incremental cost-effectiveness ratio (ICER) Quality-adjusted life-year (QALY)

The subsequent meetings were used to develop consensus around seven key themes that were identified by the steering committee and that the panel agreed needed further guidance. These themes were initially identified from a narrative literature review and were iterated on during the meetings. Previous HTAs were retrieved by conducting a targeted literature search between May 15th, 2021, and December 31st, 2021. Grey literature sources were searched using the INAHTA Database, Centre for Reviews and Dissemination of the University of York, Google Scholar, and HTA Agencies. A list of agency websites was created by cross-referencing agencies listed in INAHTA, EUnetHTA, HTAi, RedETSA, HTAsiaLink, and WHO HTA Profiles. Identified HTA reports were reviewed for any additional references to other agency websites or reports not found in the original search. HTA reports were included if they reviewed robotic-assisted, soft-tissue procedures. Further relevant articles were retrieved through snowball sampling.

Consensus was measured using a modified Delphi method with each consensus point voted on by the panel 2–3 times, with alterations to the statements made where necessary, until at least 75 percent consensus was reached. Each consensus point was discussed by the panel and then voted on to compute a level of agreement, plotted on a Likert scale. A level of agreement was computed after each discussion of the consensus statements, and alterations to the statements were permitted where agreement was not reached, or the panel felt that the wording of the statements was unclear. The combination of these methods allowed for both private voting and explicit measurement, as well as an interactive discussion to build upon high-level consensus points.

## Results

A total of ninety-eight HTAs from twenty-five different countries were identified, with Canada (*n* = 16) providing the greatest number. From the ninety-eight reports, thirty-six were sufficiently detailed on methods and results to warrant in-depth review. Of these thirty-six reports, twelve assessed prostatectomy, three hysterectomy, one thyroidectomy, two rectal resection, three partial nephrectomy, one on thoracic procedures, one on gynecological procedures, and thirteen reviewed multiple procedures.

**Figure 1.**
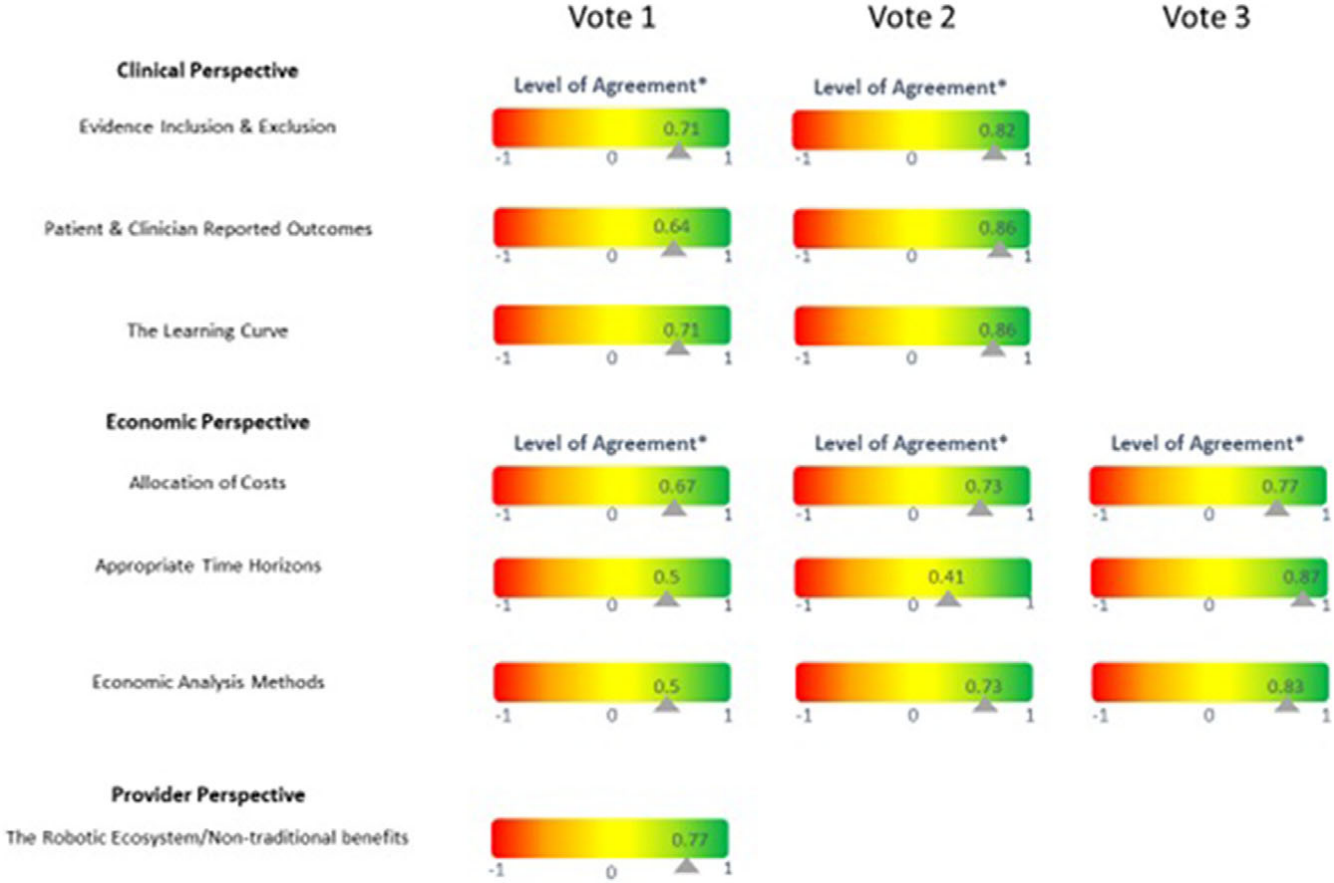
Likert scales showing level of agreement on consensus statements.

The steering committee extracted seven themes from the review of previous HTAs and generated draft consensus statements, summarized in Table 3. Each expert responded to each statement by rating their level of agreement from strongly disagree (−1), disagree (−0.5), neutral (0), agree (+0.5), and strongly agree (+1). The values shown in Figure 1 are the mean values of the responses.

**Table 3.** Themes from the current literature and consensus statements to be reached

Theme	Initial consensus point	Final consensus point
*Clinical perspective*		
Evidence inclusion and exclusion	Given the complexity in assessing the clinical effectiveness of robotic ecosystems in practice, real-world evidence should be considered for inclusion in health technology assessments	Same as the initial consensus point
Patient- and clinician-reported outcomes	In addition to outcomes measuring clinical effectiveness (such as disease-free survival and positive surgical margin), outcomes reflecting the patient and clinician perspective should also be considered by HTAs	Same as the initial consensus point
The learning curve	HTAs of RAS should take differences in clinician competency into account and where possible correct for learning curve effects	Same as the initial consensus point
*Economic perspective*		
Allocation of costs	The procedure volume used in cost models of RAS should consider all types of procedures that a hospital may use the system for	National-level HTA’s of RAS should consider robotic ecosystems on an (established) multi-therapeutic area basis, reflecting the procedural capabilities of the platform. The operating costs should be apportioned according to procedure volume and considering all established procedures that the platform can undertake
Appropriate time horizons	It is appropriate to use a 10-year time horizon when building cost models of RAS	The time horizon when building a value framework for a robotic platform should consider the clinical outcome and the level of decision maker (i.e., national vs local)
Economic analysis methods	Multi-procedure cost analysis would more accurately model the cost-effectiveness of a robotic system in practice, and therefore, methodological work is needed to develop these approaches	Same as the initial consensus point
*Provider perspective*		
The robotic ecosystem	The value of the entire robotic ecosystem should be considered by HTA’s conducted on robotic assisted surgery	N/A

HTA, health technology assessment; RAS, robotic assisted surgery.

### Key themes

The seven key themes and related consensus statements are listed below; the order does not indicate importance.

### Evidence inclusion and exclusion

*“Given the complexity in assessing the clinical effectiveness of robotic ecosystems in practice, real-world evidence should be considered for inclusion in health technology assessments.”*

The panel agreed that regardless of study design, research must be of high quality and highlighted the need to take a lifecycle approach to evidence generation (i.e., recent evidence). RWE may be more susceptible to certain biases that randomization may eliminate, but it can provide larger group sizes, more generalizability, and looks at the average impact across a wider population. The group also considered that evidence around benefits such as training and operational and organizational factors may be better collected in RWE studies than in RCTs.

### Patient and clinician perspectives

*“In addition to outcomes measuring clinical effectiveness (such as disease-free survival and positive surgical margin), outcomes reflecting the patient and clinician perspective should also be considered by HTAs.”*

The panel agreed that commonly collected clinical effectiveness measures translate to increased benefit for the patient. However, patient preference, experience, and satisfaction are also critically important outcomes for surgical interventions and the patient perspective should be a priority for future evidence generation and HTA considerations. Surgeon ergonomics and longevity should also be considered, although the panel acknowledged that these may take many years to capture the true effects.

### Learning curve effects

*“HTAs of RAS should take differences in clinician competency into account and where possible correct for learning curve effects.”*

The panel agreed that competency is an important factor when considering the learning curve and that this does not always equal a set number of procedures (37). According to the panel, competency is context specific, but that experience is, to some extent, transferable between certain robotic procedures given a surgeon’s experience on a platform. However, competency is unlikely to transfer from one platform to another, which may suggest that evaluating single platforms on a multi-procedure basis may be the appropriate approach. Further, competency is not restricted to the surgeon and should be considered for the entire surgical team.

### Allocation of costs

*“‘National-level HTA’s of RAS should consider Robotic Ecosystems on an (established) multi-therapeutic area basis, reflecting the procedural capabilities of the platform. The operating costs should be apportioned according to procedure volume and considering all established procedures that the platform can undertake.”*

RAS platforms require large up-front purchases, as well as continued disposables costs. The panel agreed that given the wide range of procedures that can be completed with a single platform, it is important that any assessment of the technology apportions those costs across all procedures.

### Analysis methods

*“Multi-procedure cost analysis would more accurately model the cost-effectiveness of a robotic system in practice and therefore, methodological work is needed to develop these approaches.”*

The panel concluded that multi-procedure assessments are most applicable to local decision making through hospital-based HTAs (hereafter HB-HTAs), whereas national assessments are more likely to focus on an individual indication/procedure.

The panel suggested a value-based healthcare (VBHC) approach may be more appropriate traditional cost-effectiveness approach and considered many methods for overcoming these difficulties. One such methodology is the time-driven activity-based costing, which is a methodology that underpins the VBHC delivery model (38). This model allows for the costing of each step a patient takes along the entire care pathway.

### Appropriate time horizons

*“The time horizon when building a value framework for a robotic platform should consider the clinical outcome and the level of decision maker (i.e., national vs local).”*

The panel agreed that the clinically relevant time horizon is likely to differ depending on the procedure, and whether the underlying disease is benign or malignant. Malignant disease may require time horizons of over 5 years, while benign disease may only require 2–4 years. Functional outcomes may only require 1-year follow-up to observe all impact while oncological outcomes may require several years. In all cases, however, the time horizon should be justified by the context.

Another possible challenge is that differences in certain clinical outcomes (e.g., recurrence) might show up a few years after surgery, but their impacts on quality of life and cost will be carried forward for much longer time. As mentioned previously, the impact on surgeon well-being may take even longer to capture.

### The robotic ecosystem and wider benefits

*“The value of the entire robotic ecosystem should be considered by HTA’s conducted on robotic assisted surgery.”*

The panel considered that robotic platforms provide additional benefits that are not commonly considered by HTAs. Other organizational benefits related to operational efficiency, data analytics, or the ability to perform tele-surgery may become more valuable in the future as the technology develops. Miniaturization is likely to be of a greater consideration for hospitals that do not have an abundance of space. Such benefits will likely be platform specific and may be best considered by HB-HTAs.

## Discussion

Robotic platforms are multi-indication tools, not therapies, and should be evaluated in this context. The panel discussed that RAS platforms are tools used by surgeons to provide a multitude of therapies, with varying clinical and cost-effectiveness measures. Therefore, individual, procedure-by-procedure assessments may not be appropriate given the multi-specialty applications of the system and the differing perspectives between types of HTAs. Evaluating the utility of a platform is not straightforward and depends on the perspective of the assessment, whether at the national or local level.

In HTAs, the societal perspective views needs and problems to solve differently than the perspectives of hospitals, both of which need to be considered in the context of how the platform will be used. Decision makers should be mindful of both perspectives.

There is a particularly strong case for RWE in the evidence generation cycle and lifecycle management of RAS. This should not diminish the utility of randomized research, rather, observational studies should complement RCTs, and the evidence base should be evaluated holistically.

As new procedures are being completed robotically and as new or existing platforms are being developed or enhanced, RWE may be generated more feasibly and avoids the need to conduct an RCT for each new procedure, platform, or modification. The circumstances, point of maturity of the technology, and procedure being studied may lend itself better to RWE than RCTs. Of course, the quality of evidence is vital. Pongiglione et al. (39) identified seventy-seven RWE sources on robotic procedures and concluded that while there is potential for applying these to HTA decision making, data accessibility, a lack of standardization of health and economic outcomes, and inadequate comparators still represent a challenge. As digital data-collection technology integrated into robotic platforms develops, this may begin to improve accessibility to high-quality real-world data. HTAs may consider how robust the data infrastructure is on a platform as a factor in their assessment and can advise robotics companies as to what data their systems should be collecting. The creation of useful registries may allow for large amounts of high-quality real-world data to be continuously generated by systems already in use. This could facilitate continuous assessment of the technology, particularly for less-established procedures where data are more limited. It should be noted, however, that if postmarket surveillance suggests that the cost-effectiveness of RAS platforms is less than previously observed, HTA bodies and policy makers may recommend for the de-implementation of these platforms. This may provide difficulties from the perspective of hospitals using the platforms and surgeons who are now trained on them. Further discussion may be required on how to deal with this potential issue.

While PROs have been captured in some studies of RAS, evidence on clinician-reported outcomes or clinician benefits is far less common. Surgeon longevity is a benefit, particularly in settings where recruitment and retention of surgeons are low. However, it should be acknowledged that this is not a straightforward outcome to capture, and importance varies with health system structure and across societal values, particularly in certain countries or regions where surgeon benefit may be highly valued.

Surgeon fatigue and ergonomics have been captured in previous studies (40), but more data are required to allow HTAs and decision makers to factor such outcomes into their conclusions. RWE may be particularly useful for capturing such outcomes. Ideally, downstream effects of such outcomes should be quantified. This potentially could be captured through reduction in surgical mistakes, increased efficiency metrics, or reduction of surgeon injuries. Recruitment and retention of surgeons, however, are an organizational factor that is not currently considered by most national HTAs. There are exceptions, such as the Netherlands, which does consider organizational impacts in HTA processes (41).

HB-HTA may place more of an emphasis on this aspect of the technology if recruitment is a factor in their decision making (42). Even from a national perspective, this is a significant consideration as surgeon shortages are predicted to grow (43).

Hospital-level purchasing decisions are currently more likely to be affected by surgeon-reported outcomes than at national level where patient factors are considered in current frameworks; however, the needs of both perspectives should be acknowledged by HTAs. Future research should also highlight these measures and aim to standardize methodologies for collecting them. The ongoing RoboCOS study (44) may be a potential solution to this issue.

Each RAS procedure will have different clinical effectiveness and therefore a different cost-effectiveness. However, each hospital will use the platform for a casemix of different procedures and volumes. This has contributed to varying estimates of cost-effectiveness. Additionally, HTAs that do not account for all procedures risk overestimating the fixed cost per procedure. A potential solution to this issue may be national (or regional) HTAs calculating a minimum procedure volume threshold for established procedures, to be cost-effective. HB-HTAs or decision makers can then assess the viability of using the platform from a utilization standard point based on their casemix. This approach may be most appropriate given that the system is shared across different procedures with varying adoption rates. For instance, there is a higher likelihood surgeons will pass their learning curve in established procedures and that more robust evidence exists to support an HTA. Allowing hospitals to use the platform for other less-established procedures will also contribute to RWE generation across the system lifecycle to continuously evaluate clinical and economic outcomes of emerging technology and newer procedures.

Additionally, HTAs that allocate the entire cost of the system to one procedure misrepresent how the tool is utilized in practice. Capital costs of the system can be distributed to a certain procedure volume of a single procedure, based on proportionality of intended procedure volume. For example, if it is estimated that robotic prostatectomy accounts for 70 percent of an average hospital’s procedure volume, then 70 percent of the overall cost may be considered in a national HTA of robotic prostatectomy. The remainder can be allocated to other procedures within the cost structure. This fraction may be varied in sensitivity analysis.

Difficulties also arise when assessing a technology across multiple indications using standard HTA methods as the outcomes and comparators will differ. A recent paper by Patel et al. (45) attempted to develop an online interactive model for calculating the value for money of a specific robotic procedure. This model may be a potential solution that can be easily implemented at hospital level.

Previous HTAs have used a wide variety of time horizons with longer time horizons generally provided more favorable results (see Supplementary Table 2). However, longer time horizons increase uncertainty. Guidelines on appropriate time horizons from HTA bodies vary across the world, although most countries give broadly similar advice (19;35). The International Consortium for Health Outcomes Measurement standard set for advanced prostate cancer sets out a maximum required follow-up of 3 years for treatments and adverse events, PROs, and disease-free survival (46;47). While longer time horizons may capture certain outcomes, such as surgeon longevity over periods of 10 years, there is a requirement for long-term postmarket surveillance studies to capture these outcomes. Wider benefits that are derived from the ecosystem surrounding the robotic platform should also be considered. HTAs should aim to consider the benefits of technologies like data analytics, training through simulation, and 3D visualization of anatomy in a more holistic fashion.

### Future outlook

The recommendations discussed in this paper may be transferable to other surgical types. Orthopedic and neurosurgery platforms are also likely to be used for a variety of different procedures across different therapeutic areas and diseases and have similarly high capital costs.

It is difficult to determine a cadence of time for which these recommendations should be revisited to consider a rapidly evolving technological field. Greater incorporation of artificial intelligence, increased levels of robotic autonomy, and continued integration of advanced digital components may be a catalyst for reconsidering the topic.

### Limitations

While this work discussed in depth the challenges associated with assessing RAS and suggested some best practices, it did not engage in any methodological development. The panel acknowledged the ongoing work of the RoboCOS study (43) into the development of a core set of outcomes for RAS and awaits the results of this study. Further, the panel suggested that further methodological work into multi-procedure cost-effectiveness analysis should be undertaken.

This study aimed to gather the expert inputs of relevant stakeholders in the assessment of RAS. Despite gathering broad expertise, there were limitations to this approach. Any expert panel produces only subjective results, and this panel included only fifteen members. A standard Delphi methodology including a larger number of experts could have been employed to widen the range of results; however, it was considered that depth of discussion was vital. To account for the lack of responses, the group met four times in total (as opposed to a single meeting in a standard Delphi approach). A further limitation the panel acknowledged is a lack of patient representation. It was considered difficult to include a patient representative with the relevant experience and knowledge to add to the expert panel discussion. Further work may look to discuss the results of the panel discussion with patients to gain a vital perspective on the value of RAS. In particular, patient perspectives on the recommendations regarding patient-related outcomes and the potential societal benefits of RAS would be invaluable.

## Conclusions

Robotic surgical platforms are tools that provide multi-indication and multi-stakeholder value beyond what is usually assessed in a therapeutic HTA perspective. Their value should be considered across multiple therapeutic areas, which requires some new contemporary methodological considerations for HTAs.

Differences in perspective between national and hospital-level HTA led to most of the contention between the panels. Both national and local HTAs have a distinct role to play in the assessment of RAS platforms, as do both RCTs and RWE. The list of principles set out in this paper should help HTA bodies at all levels to evaluate RAS.

A high-level framework has been developed here, but this work may be expanded with more methodological research. Recommendations made here should be transferable to other types of surgical systems and other disciplines. Further, this work may serve as a first case study for swiftly developing areas in MedTech that require consideration for HTAs. Digital technologies, in particular, may benefit from similar discussions that result in standards, guidance, or recommendations for appropriate evaluation.

## Supplementary material

For supplementary material accompanying this paper visit https://doi.org/10.1017/S0266462323000314. click here to view supplementary material
