# Supplementary material for: Best practice considerations on the assessment of robotic assisted surgical systems: results from an international consensus expert panel
Source: Int J Technol Assess Health Care. 2023 Jun 5;39(1):e39. doi: 10.1017/S0266462323000314 (PMC11570098; doi:10.1017/S0266462323000314)
Supplement: Supplementary file 1 [file S0266462323000314sup.zip › S0266462323000314sup002.docx]

Supplementary Table 1 - Inclusion and Exclusion Criteria from HTAs of robotic prostatectomy (and others)

| **HTA Body/Author** | **Country, Date** | **Comparators** | **Inclusion Criteria** | **Conclusions from Clinical Evidence** |
| --- | --- | --- | --- | --- |
| University of Alberta | Canada, 2017 | Open & Laparoscopic Prostatectomy, radiation therapy and cryoablation | Randomized and non-randomized controlled trials; Cohort studies; Case-control studies | Improved oncological, functional and perioperative outcomes compared with open surgery.  Comparable oncological, functional and perioperative outcomes compared with laparoscopic surgery. |
| HIQA | Ireland, 2011 | Open & Laparoscopic Prostatectomy & Hysterectomy | Systematic reviews; meta-analyses; technology assessments; randomized controlled trials; controlled clinical trials; observational studies, clinical practice guidelines | Improved oncological, functional and perioperative outcomes compared with open surgery.  Comparable oncological, functional and perioperative outcomes compared with laparoscopic surgery. |
| University of Calgary | British Columbia, Canada, 2021 | Open & Laparoscopic Prostatectomy | Previous HTAs and Clinical Guidelines |  |
| NIHR | UK/England 2012 | Open & Laparoscopic Prostatectomy | Randomized Controlled Trials and non-randomized comparative studies.  Case-series for estimates of learning curve effects, only |  |
| Health Quality Ontario | Canada, 2017 | Open & Laparoscopic Prostatectomy | Randomized controlled trials (RCTs), prospective comparative nonrandomized studies, and systematic reviews | Reduced length of stay and blood loss (moderate quality evidence) but had no difference or inconclusive results for functional and oncological outcomes (low to moderate quality evidence). Compared with laparoscopic radical prostatectomy, robot-assisted radical prostatectomy had no difference in perioperative, functional, and oncological outcomes (low to moderate quality evidence). |
| Swiss Medical Board | Switzerland, 2018 | Open & Laparoscopic Prostatectomy & Hysterectomy | Previous Cochrane reviews of RCTs | Do not report clinical outcomes. |
| CONITEC – National Committee for Technology Incorporation | Brazil, 2018 | Open & Laparoscopic Prostatectomy | Previous HTAs, Systematic reviews, and RCTs | RAS has some potential benefits related to blood loss, transfusions, and sexual function. Other benefits pointed out include urinary continence, and LOS due to lower rates of perioperative complications. However, given the wide confidence intervals presented and the low quality of studies, it is not possible to conclusively state that there are statistically significant benefits in favor of the intervention. |
| HAS | France, 2016 | Open & Laparoscopic Prostatectomy | **Previous HTAs, Systematic reviews w meta-analyses, and RCTs.** Prospective comparative studies after 2013 considered where RCTs were not available. Observational studies with n>100 considered for data on perioperative complications | **Open:** No conclusion made on overall survival, and on functional and oncological outcomes.  Reduced risk of blood loss. No conclusion on risk of blood transfusion.  **Laparoscopic:** No conclusion made on overall survival, and on functional and oncological outcomes.  No conclusion on risk of blood loss or blood transfusion |
| National Centre for Health Technology Excellence | Mexico, 2019 | Open & Laparoscopic Prostatectomy | Systematic Reviews | No significant differences in oncological outcomes or sexual and urological function.  Reduction in blood loss and need for blood transfusion. |
| HIQA: Health Information and Quality Authority; HAS: Haute Autorité de santé; HTA: Health Technology Assessment; LOS: Length of Stay; NIHR: National Institute for Health Research; RAS: Robotic Assisted Surgery; RCT: Randomised Controlled Trial | | | | |
| References (in order as above)  Health Technology & Policy Unit, University of Alberta (2017) “Robot-Assisted Laparoscopic Prostatectomy (RALP)”. Available at: <https://open.alberta.ca/publications/robot-assisted-laparoscopic-prostatectomy-ralp-final-report>  Health Information and Quality Authority (2011) “Health technology assessment of robot-assisted surgery in selected surgical procedures”. Available at: <https://www.hiqa.ie/reports-and-publications/health-technology-assessment/hta-robot-assisted-surgery>  British Columbia Health Technology Assessment (2021) “Open Radical Prostatectomy, Laparoscopic Radical Prostatectomy, and Robotic-assisted Radical Prostatectomy”. Available at: <https://www2.gov.bc.ca/assets/gov/health/about-bc-s-health-care-system/heath-care-partners/health-authorities/bc-health-technology-assessments/prostatectomy-hta.pdf>  Ramsay, C. et al. (2012) “Systematic review and economic modelling of the relative clinical benefit and cost-effectiveness of laparoscopic surgery and robotic surgery for removal of the prostate in men with localised prostate cancer.,” Health Technology Assessment, 16(41). Available at: <https://doi.org/10.3310/hta16410>.  Health Quality Ontario (2017) “Robotic surgical system for radical prostatectomy: a health technology assessment”. Ont Health Technol Assess Ser [Internet]. Available at: <http://www.hqontario.ca/evidence-to-improve-care/journal-ontario-health-technology-assessment-series>  Swiss Medical Board (2018) “Robot-assisted versus open surgery for radical prostatectomy. Robot-asssited versus laparoscopic surgery for simple or radical hysterectomy”. Available at: <https://www.swissmedicalboard.ch/fileadmin/public/news/2018/assessment_report_smb_robot-assisted_surgery_long_2018.pdf>  National Committee for Health Technology Incorporation in the Sistema Unica Saude (2018) “Robotic Surgical System for Minimally Invasive Surgery: Radical Prostatectomy”. Available at: <https://www.gov.br/conitec/pt-br/midias/relatorios/2018/recomendacao/relatorio_davinci_prostatectomia.pdf>  HAS (2016) “Évaluation des dimensions clinique et organisationnelle de la chirurgie robot-assistée dans le cadre d’une prostatectomie totale”. Available at : <https://www.has-sante.fr/jcms/c_2037513/fr/evaluation-des-dimensions-clinique-et-organisationnelle-de-la-chirurgie-robot-assistee-dans-le-cadre-d-une-prostatectomie-totale>  National Center for Health Technology Excellence. “Clinical and Economic Evaluation of Radical Surgery Alternatives for the Treatment of Localized Prostate Cancer”. 2019. <https://www.gob.mx/cms/uploads/attachment/file/630060/EC_EE_AlternativasDeCirug_aRadicalCapros_FINAL.pdf> | | | | |
