# Supplementary material for: Best practice considerations on the assessment of robotic assisted surgical systems: results from an international consensus expert panel
Source: Int J Technol Assess Health Care. 2023 Jun 5;39(1):e39. doi: 10.1017/S0266462323000314 (PMC11570098; doi:10.1017/S0266462323000314)
Supplement: Supplementary file 1 [file S0266462323000314sup.zip › S0266462323000314sup003.docx]

**Supplementary Table 2 - Comparison of Economic Methodologies from 4 HTAs of Robotic Prostatectomy**

|  | **HQO, Canada (2017)** | **Parackal, Canada (2020)** | **HIQA, Ireland (2011)** | **Australia (2006)** |
| --- | --- | --- | --- | --- |
| Time Horizon | 1 year | 10 years | 7 years (median) | 3 years |
| Source of Effectiveness Parameters | Single RCT (Yaxley et al. 2016) | SLR and Meta-Analysis of observational comparative studies | Expert Advisory Group, SLR with meta-analysis, data collection | Literature review (n=7) |
| Discount Rate (for Cost and QALYs) | 5% | 1.5% | 4% | 5% |
| Allocation of Capital Cost* | (Equivalent annual cost + annual service fee) ÷ annual procedure volumes | (Equivalent annual cost of system/10 years + annual maintenance cost) ÷ annual procedure volumes | Initial capital cost followed by 7% interest per annum + maintenance costs | Linear depreciation over equipment life span with a residual value of zero. Maintenance costs 5-10% of system costs. |
| Service Life Span of System** | 9 years | 10 years | 7 years | 7 years |
| Annual Procedure Volumes | 200 | 400 | Prostatectomy 200; Hysterectomy 300; Combined 300 | 300 |
| Cost Per Procedure for RAS (base case) | $5,265 (CAD) | $1,930 (CAD) | £3,701 (GBP) (~6000CAD) | €2709 (AUD) (~2,500CAD) |
| Expected Cost over time horizon (base case results) | $20,604CAD (1 year) | $47,033 (CAD) (over 10 years) | Several | $17,562.00 (AUD) (~16,500 CAD) |
| Incremental Cost vs Open | $6,234 (CAD) (1 year) | $1,701 (CAD) (over 10 years) | €2,487.00 (EUR) (~3600CAD) | $4,502 (AUD) (~4250CAD) |
| Expected QALYs over time horizon (base case results) | 0.9296 | 7.2047 | Not Reported | 6.93 |
| Incremental QALYs vs Open | 0.0012 | 0.0662 | 0.093 | 0.1 |
| **ICER (base case results)** | **$5,200,000 (CAD)/ QALY** | **$25,704 (CAD)/ QALY** | **€26,647 (EUR)/QALY (~38,725CAD)/QALY)** | **$450,200 (AUD) (~425,000CAD)/QALY** |
| AUD: Australian Dollar; CAD: Canadian Dollar; EUR: Euro; GBP: Great British Pound; HQO: Health Quality Ontario; ICER: Incremental Cost Effectiveness Ratio; QALY: Quality-Adjusted Life Year; RCT: Randomised Controlled Trial; SLR: Systematic Literature Review  Exchange Rates Correct as of 02 Feb 2023 | | | | |
| Health Quality Ontario (2017) “Robotic surgical system for radical prostatectomy: a health technology assessment”. Ont Health Technol Assess Ser [Internet]. Available at: <http://www.hqontario.ca/evidence-to-improve-care/journal-ontario-health-technology-assessment-series>  Parackal, A. et al. (2020) “Economic evaluation of robot-assisted radical prostatectomy compared to open radical prostatectomy for prostate cancer treatment in Ontario, Canada,” Canadian Urological Association Journal, 14(8). Available at: <https://doi.org/10.5489/cuaj.6376>.  Health Information and Quality Authority. (2011) “Health technology assessment of robot-assisted surgery in selected surgical procedures”. Available at: https://www.hiqa.ie/reports-and-publications/health-technology-assessment/hta-robot-assisted-surgery  Medical Services Advisory Committee. (2006) “Laparoscopic Remotely Assisted Radical Prostatectomy”. Available at: <http://www.msac.gov.au/internet/msac/publishing.nsf/Content/1091-public>  Yaxley, J.W. et al. (2016) “Robot-assisted laparoscopic prostatectomy versus open radical retropubic prostatectomy: Early outcomes from a randomised controlled phase 3 study,” The Lancet, 388(10049), pp. 1057–1066. Available at: https://doi.org/10.1016/s0140-6736(16)30592-x. | | | | |
