# Supplementary figures and images for: Best practice considerations on the assessment of robotic assisted surgical systems: results from an international consensus expert panel
Source: Int J Technol Assess Health Care. 2023 Jun 5;39(1):e39. doi: 10.1017/S0266462323000314 (PMC11570098; doi:10.1017/S0266462323000314)

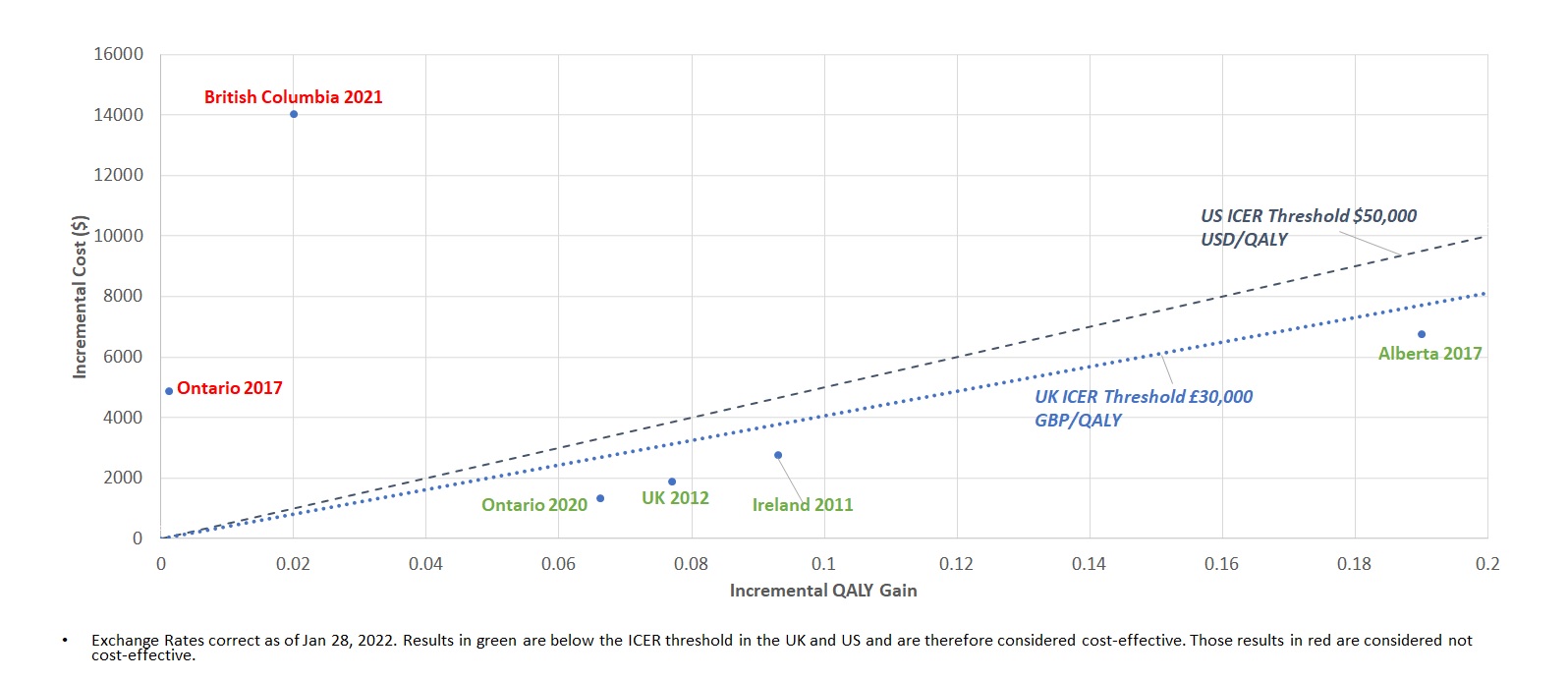

Supplement: Supplementary file 1 [file S0266462323000314sup.zip › S0266462323000314sup001.jpg]
